# Preparedness for Coronavirus Disease in Hospitals of Nepal: A Nationwide Survey

**DOI:** 10.31729/jnma.4941

**Published:** 2020-04-30

**Authors:** Gentle Sunder Shrestha, Hem Raj Paneru, Subhash Prasad Acharya, Sanjeet Krishna Shrestha, Mahesh Raj Sigdel, Sanjeeb Tiwari, Bharat Kumar Yadav, Badri Rijal, Lochan Karki, Yogesh Neupane, Narmaya Thapa, Sanjay Lakhey

**Affiliations:** 1 Department of Anaesthesiology, Tribhuvan University Teaching Hospital, Kathmandu, Nepal; 2 Department of Pulmonary, Critical Care and Sleep Medicine, Nepal Mediciti Hospital, Lalitpur, Nepal; 3 Department of Nephrology, Tribhuvan University Teaching Hospital, Kathmandu, Nepal; 4 Department of General Practice and Emergency Medicine, Tribhuvan University Teaching Hospital, Kathmandu, Nepal; 5 Department of General Practice and Emergency Medicine, Patan Academy of Health Sciences, Lalitpur, Nepal; 6 Department of Orthopaedics, National Trauma Center, Kathmandu, Nepal; 7 Department of Internal Medicine, National Academy of Medical Sciences, Bir Hospital, Kathmandu, Nepal; 8 Department of ENT and Head & Neck Surgery, Ganesh Man Singh Memorial Academy of ENT - Head & Neck Studies, Tribhuvan University Teaching Hospital, Kathmandu, Nepal; 9 Department of Internal Medicine, B and B Hospital Pvt Ltd, Lalitpur, Nepal

**Keywords:** *COVID-19*, *hospital preparedness*, *Nepal*

## Abstract

**Introduction:**

Coronavirus disease (COVID-19) pandemic has affected large number of people globally and has continued to spread. Preparedness of individual nations and the hospitals is important to effectively deal with the surge of cases. We aimed to obtain nation wide data from Nepal, about hospital preparedness for COVID-19.

**Methods:**

Online questionnaire was prepared in accordance with the Center for Disease Control recommendations to assess preparedness of hospitals for COVID-19. The questionnaire was circulated to the over 800 doctors across the nation, who are the life members of six medical societies.

**Results:**

We obtained 131 completed responses from all seven provinces. Majority of respondents had anaesthesiology as the primary specialty. Only 52 (39.7%) participants mentioned that their hospital had policy to receive suspected or proven cases with COVID-19. Presence of isolation ward was mentioned by 83 (63.4%) respondents, with only 9 (6.9%)mentioning the presence of airborne isolation. Supply of personal protective equipment (PPE) was inadequate as per 124 (94.7%) respondents. Critical care services for COVID-19 patients were possible only in hospitals of 42 (32.1%)respondents. RT-polymerase chain reaction could be performed only in the hospital of 6 (4.6%) respondents.

**Conclusions:**

It is apparent that most of the hospitals are not well prepared for management of patients with COVID-19. Resource allocation and policy making should be aimed to enhance national preparedness for the pandemic.

## INTRODUCTION

As of April 20, 2020, over 2.3 million people are affected by Coronavirus Disease (COVID)-19, with over 157,000 deaths and involving over 200 nations.^1^ Around 81% of the patients have mild illness, 14% develop severe disease requiring hospitalization and 5% become critically ill.^2^ Nepal has 31 confirmed cases of COVID-19, with 111 patients under isolation, as of April 20, 2020.^3^

The speed of spread of disease is breath taking. The existing health care resources can be rapidly overwhelmed as the nations observe the surge in cases, more so in the countries with limited resources like Nepal. It is imperative to assess the level of preparedness of the nation, before being hit hard by the pandemic. We aimed to obtain nation-wide data about hospital preparedness for COVID-19.

## METHODS

This is a descriptive cross-sectional study based on an online questionnaire prepared using google form, which is in accordance with the Center for Disease Control (CDC) recommendations to assess preparedness of hospitals for COVID-19.^4^ The questionnaire was circulated to the doctors across the nation, involving all 7 provinces, and over the duration of a week. Questionnaire were mailed to over 600 doctors, who are the life members of six societies - Nepalese Society of Critical Care Medicine, Society of Anaesthesiologists of Nepal, Society of Internal Medicine of Nepal, General Practitioner Association of Nepal, Society of Otolaryngologists of Nepal and Nepal Medical Association. They were the sampling population of the study.Convenience sampling was done and the sample size was calculated using the formula

$$\begin{array}{ll} \text{Sample size }(\text{n)} & \text{= }{\text{Z}}^{\text{2}}\times \text{p }\times \text{q}/{\text{e}}^{\text{2}}\\ & \text{= 1}{\text{.96}}^{\text{2}}\times 0.5\times 0.5/{\left(0.05\right)}^{\text{2}}\\ & \text{= }384 \end{array}$$

Total known study population (N): 600
Adjusted Sample size: n/ 1 + n - 1/N
= 234
where z = 1.96, at the confidence interval of 95%;
prevalence is assumed to be 50%, q = 1-p
and e = 5%.

Considering the non-response rate of 15%, sample size of 269 was calculated. Selection bias was possible, as only the doctors were enrolled in the survey. Other medical personnel, who can be involved in the management of COVID-19 patients were not enrolled. Participation in the survey was voluntary. It was mentioned in the beginning of the survey that, by participating in the survey, the participant would be agreeing to consent the use of data from the survey for the purpose of publication and for possible future national policy making. Ethical approval was not obtained, considering the urgency and time sensitive nature of the survey, and the survey being non-interventional. However the study has followed the principles of ethics stated by Declaration of Helsinki, developed by World Medical Association.^5^ The survey imposed no harm to the participants, also, the vulnerable groups and individuals were not involved in the survey. The dignity, integrity, right to self-determination, privacy, and confidentiality of personal information of the participants were maintained.^5^ We obtained 131 completed responses over the duration of a week (April1 to 7, 2020). The duration of study was pre-determined and considering the less likelihood of receiving further responses and the need to conduct the survey promptly, study was terminated before reaching the calculated sample size.

## RESULTS

**Figure 1.**
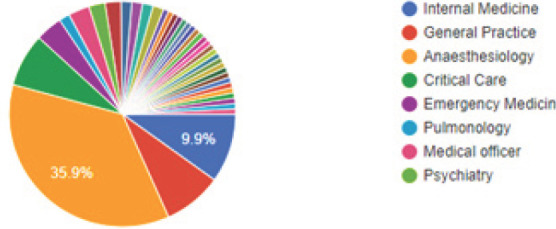
Primary specialty of respondents.

**Figure 2.**
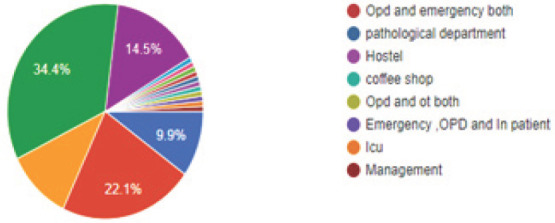
Workplace distribution (where respondents spend most of their time).

We obtained responses from all provinces (69 from province 3, 28 from province 1, 10 from province 2, 9 from province 5, 7 from province 7, 5 from province 4 and 3 from province 6). Of the respondents, 47 (35.9%) had anaesthesiology as their primary specialty (Figure 1), with 45 (34.4%) respondents working in operating room (Figure 2).

**Figure 3.**
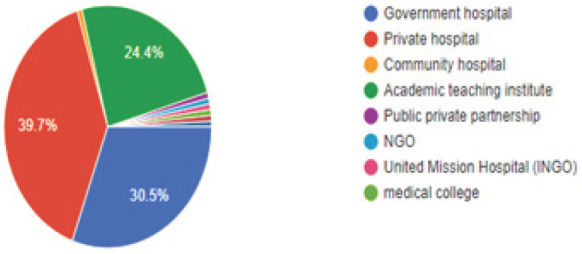
Category of hospitals (where the respondents work in).

Most of the respondents, 52 (39.7%) worked in the private hospital, followed by 40 (30.5%)working in government hospital and 32 (24.4%)working in academic teaching institute (Figure 3).

The summary of responses from the participants of the survey is shown (Table 1).

**Table 1:** Summary of responses from the participants of the survey

Question	Response	
	Yes n(%)	No n(%)
Education and training program on COVID-19	73 (55.7)	58 (44.3)
Designated triage area in hospital	70 (53.4)	62 (47.3)
Defined triage policy	71 (54.2)	60 (45.8)
Process to notify suspected or proven COVID-19	96 (73.8)	34 (26.2)
Hospital policy or process for receiving suspected or proven cases of COVID-19	52 (39.7)	79 (60.3)
Hospital policy for referring suspected or proven cases of COVID-19	82 (62.6)	49 (37.4)
Patient isolation policy	81 (61.8)	50 (38.2)
Availability of isolation ward	83 (63.4)	48 (36.6)
Availability of airborne isolation room	9 (6.9)	122 (93.1)
Sufficient supply of PPE	7 (5.3)	124 (94.7)
Sufficient provision of sanitizers/handwash	86 (65.6)	45 (34.4)
Established process of audit	34 (26)	97 (74)
Hospital policy for monitoring and managing healthcare workers with exposure to COVID-19	27 (20.6)	104 (79.4)
Hospital policy for visitors of patients with suspected COVID-19	26 (20)	105 (80)
Hospital planning to deal with possible surge	44 (33.6)	87 (66.4)
Can your hospital provide critical care service to COVID-19	42 (32.1)	89 (67.9)
Does your hospital perform RT-PCR for COVID-19	6 (4.6)	125 (95.4)
Can your hospital do CT chest?	76 (58)	55 (42)

Education and training program for COVID-19 was present in only 73 (55.7%) hospitals. Only 52 (39.7%) participants mentioned that their hospital had policy to receive suspected or proven cases with COVID-19. Isolation policy was present in the hospitals of 81 (61.8%) respondents and 83 (63.4%)mentioned the availability of isolation ward. However, 63 (48.1%) respondents had limited number of (<10) isolation beds in their hospital. Presence of airborne isolation room was reported by only 9 (6.9%) respondents. Supply of personal protective equipment (PPE) was inadequate as per 124 (94.7%) respondents. Established auditing mechanism for infection prevention and control program was present only in 34 (26%) respondents' hospital. Only a few hospitals had policy for monitoring and managing the health care workers with exposure to COVID-19. Presence of hospital planning to deal with possible surge in cases was mentioned by 44 (33.6%) respondents. Critical care services for COVID-19 patients were possible only in hospitals of 42 (32.1%) respondents. Level II ICU beds were present in the hospital of 38 (29%) respondents and level III beds were present in 31 (23.7%). RT-polymerase chain reaction could be performed only in the hospital of 6 (4.6%)respondents. Respondents expected support from the government in various areas to combat COVID-19 like: provision of personal protective equipment and sanitizers, team build up and training on patient management as well as training on self protection of medical staff, increasing diagnostic test capacity, providing the health care worker with guidelines on screening, isolation and treatment, allocating designated hospitals for management of COVID-19 patients, and increasing level 2 and level 3 ICU bed capacity. Moreover, respondents suggested expanding programs to raise awareness amongst public about COVID-19 with educational activities covering disease dynamics, symptoms and signs of the disease, preventive measures, and the importance of self-isolation and quarantine.

## DISCUSSION

Our survey revealed various aspects of health care in the hospitals of Nepal that need improvements to face the possible future surge in the cases. Hospitals need to develop policy to receive and manage proven or suspected patients with COVID-19 and be prepared for possible surge. There should be adequate preparation for patient isolation, with the provision of adequate isolation beds, including negative pressure isolation rooms. There is significant scarcity of ICU beds. Capacity building needs to be done to make provision of ICU beds, including level III beds to manage patients with multiorgan failure (common in patients with severe disease).^5,6^ RT-polymerase chain reaction testing need to be made available in multiple centers to facilitate rapid testing, that would indirectly help to preserve scarce resources. Secondary transmission of disease in the hospital setting has been documented. Health care workers are at increased risk or acquiring infection while treating these patients.^2^ There is significant short supply of PPE. Majority of hospitals do not have policy for monitoring and managing the health care workers exposed to the patients. There should be the provision of proper supply of PPE. Misuse and inappropriate use of PPE should be discouraged. Training and education about various aspects of COVID-19 need to be conducted by individual centers, such as hand hygiene, proper donning and doffing of PPE, proper use of respirators, etc.^7^ Safety of health care workers need to be a major priority.^8^ National bodies need to work together to come up with national guidelines tailored to local resources.

There are several limitations of the survey. Though we obtained responses from all seven provinces, not all the provinces were equally represented. The survey was limited to the life members of five medical societies, most of them being the post MD doctors. The junior doctors and other medical professionals, who are directly involved in management of patients with COVID-19 were not included. A relatively small number of responses obtained may limit the generalizability of the findings to all the hospitals of the nation. A larger study, that would equally represent all the provinces and all the categories of the hospitals and that would also include health care personnel, other than doctors, would be beneficial.

## CONCLUSIONS

This nation wide survey reveals that most of the hospitals are not well prepared for management of patients with COVID-19. Considering the limited resources of the nation, the results of this survey may help to guide appropriate resource allocation and policy making, aiming to enhance national preparedness for the pandemic.
